# Non-invasive score identifies ultrasonography-diagnosed non-alcoholic fatty liver disease and predicts mortality in the USA

**DOI:** 10.1186/s12916-014-0154-x

**Published:** 2014-09-10

**Authors:** Ching-Lung Cheung, Karen SL Lam, Ian CK Wong, Bernard MY Cheung

**Affiliations:** Department of Medicine, Li Ka Shing Faculty of Medicine, The University of Hong Kong, Pokfulam, Hong Kong; Research Centre of Heart, Brain, Hormone and Healthy Aging, Li Ka Shing Faculty of Medicine, The University of Hong Kong, Pokfulam, Hong Kong; Department of Pharmacology and Pharmacy, Li Ka Shing Faculty of Medicine, The University of Hong Kong, Pokfulam, Hong Kong; Centre for Genomic Sciences, Li Ka Shing Faculty of Medicine, The University of Hong Kong, Pokfulam, Hong Kong; The State Key Laboratory of Pharmaceutical Biotechnology, Li Ka Shing Faculty of Medicine, The University of Hong Kong, Pokfulam, Hong Kong

**Keywords:** NAFLD, NAFLD liver fat score, Mortality

## Abstract

**Background:**

Several non-invasive prediction scores for non-alcoholic fatty liver disease (NAFLD) have been developed, but their performance has not been compared and validated in the same population, and whether these prediction scores can predict clinical outcomes remains unknown. In this study, we aimed to validate and compare the performance of four NAFLD prediction scores: fatty liver index, hepatic steatosis index, lipid accumulation product, and NAFLD liver fat score (LFS), and to evaluate the ability of the best NAFLD prediction score to predict mortality.

**Methods:**

We analyzed data from the National Health and Nutrition Examination Survey conducted in 1988 to 1994, and subsequent follow-up data for mortality up to December 31, 2006. NAFLD was defined by ultrasonographic detection of hepatic steatosis in the absence of other known liver diseases.

**Results:**

In a group of 5,184 participants, LFS consistently showed the highest area under the curve for predicting the presence of NAFLD. During a median follow-up of 14.7 years (range 0.1 to 18.2 years) and 83,830.5 person-years, participants in the high LFS group (LFS ≥1.257) had a higher cardiovascular and liver-related mortality than participants in the low (LFS ≤ −1.413; cardiovascular hazard ratio (HR) = 2.24, 95% CI 1.03 to 4.88; liver HR = 31.25, 95% CI 3.13 to 333.33) or intermediate (−1.413 < LFS < 1.257; cardiovascular HR = 2.3, 95% CI 1.19 to 4.48; liver HR = 30.3, 95% CI 4 to 250) LFS groups in the fully adjusted model. Similar results were obtained when LFS was treated as a continuous variable.

**Conclusions:**

LFS is the best non-invasive prediction score for NAFLD, and people with a high LFS score have an increased risk for cardiovascular and liver-related mortality.

**Electronic supplementary material:**

The online version of this article (doi:10.1186/s12916-014-0154-x) contains supplementary material, which is available to authorized users.

## Background

Non-alcoholic fatty liver disease (NAFLD) represents a spectrum of progressive liver disease ranging from simple steatosis to non-alcoholic steatohepatitis (NASH), fibrosis, and cirrhosis, in the absence of excessive alcohol consumption. NAFLD is regarded as a hepatic manifestation of metabolic syndrome (MetS) [1], therefore the presence of NAFLD is not only strongly associated with liver-related mortality, but also with diseases related to the MetS, such as diabetes and cardiovascular diseases [2,3]. As NAFLD is highly prevalent and affects up to 30% of the general adult population [4], screening for and diagnosing NAFLD has become an important issue in public health to prevent NAFLD-related complications and reduce healthcare costs.

Liver biopsy remains the “gold standard” for NAFLD diagnosis; however, this is an invasive technique making it impractical to be used widely. Ultrasonography is therefore the recommended first-line imaging technique in clinical practice, although it is known to have limited sensitivity [3]. Other non-invasive tools have been developed for diagnosing NAFLD, such as computed tomography and proton magnetic resonance spectroscopy (^1^H-MRS). However, these tools are expensive and time-consuming, and are not considered cost-effective for large-scale NAFLD screening. Recently, five biomarker-based non-invasive prediction scores of NAFLD have been developed: SteatoTest [5], fatty liver index (FLI) [6], NAFLD liver fat score (LFS) [7], lipid accumulation product (LAP) [8], and hepatic steatosis index (HSI) [9]. These scores are derived from simple clinical risk factors and biomarkers, and can therefore potentially be used for large-scale NAFLD screening. However, different definitions and techniques were used to define NAFLD in the original studies, and the performances of these scores have not been validated, evaluated, and compared directly in a large general population. In addition, whether these non-invasive scores of NAFLD can predict clinical outcome remains largely unknown.

In this study, we aimed to validate and evaluate the performance of these non-invasive prediction scores of NAFLD in predicting ultrasonography-diagnosed NAFLD in a representative general adult population in the USA (cross-sectional NAFLD prediction cohort), and to test if the marker can predict mortality in the general population (prospective mortality prediction cohort).

## Methods

### Participant recruitment

Data from the third National Health and Nutrition Examination Survey (NHANES III) were used [10]. NHANES III was conducted by the National Center for Health Statistics (NCHS) from 1988 to 1994, using a stratified multistage probability sample that represented the civilian non-institutionalized population in the USA. Participants gave written consent before participation, and ethics approval was obtained from the Human Subjects Committee of the US Department of Health and Human Services.

We studied people aged 20 to 74 years who participated in the NHANES III survey. Laboratory tests were carried out in a mobile examination center (n = 14,797) (see Additional file 1: Figure S1). Because all non-invasive score formulae require levels of biomarkers in fasting blood, we included participants with blood taken after fasting for at least 8 hours fasting (n = 9,268). Of those, participants with factors that can confound the diagnosis of NAFLD (including excessive alcohol consumption, defined as >21 drinks/week in men and >14 drinks/week in women [4]; viral hepatitis, defined as positive serum hepatitis B surface antigen and positive serum hepatitis C antibody; iron overload, defined as transferrin saturation ≥50%; or pregnancy) were excluded (n = 1,089). The LFS formula includes fasting insulin level, therefore we further excluded participants who were using insulin or other medications for diabetes (n = 315). This left 7,864 participants, and once the appropriate exclusion criteria were adopted for each aim (see Additional file 1: Figure S1), we had 5,184 and 5,892 participants included in the analysis of Aim 1 (evaluation of the performance of non-invasive prediction scores of NAFLD in predicting ultrasound-diagnosed NAFLD) and Aim 2 (evaluation of the relationship between non-invasive prediction scores of NAFLD and mortality), respectively.

### Definition of NAFLD

In the original NHANES III between 1988 and 1994, gall bladder ultrasonography video images were recorded using a Toshiba Sonolayer SSA-90A and Toshiba video recorder. Between 2009 and 2010, hepatic steatosis (fatty liver) was assessed by archived video images being re-reviewed by three ultrasonography readers (trained by a board-certified radiologist specializing in hepatic imaging), who graded the presence of fat within the hepatic parenchyma.

The following information was recorded on a standard paper collection form: 1) presence of liver-to-kidney contrast; 2) degree of the brightness of the liver parenchyma; 3) presence of deep beam attenuation; 4) presence of echogenic walls in the small intrahepatic vessels, and (5) definition of the gallbladder walls. Finally, an overall primary finding was given based on the presence or absence of each of the five parameters. The liver was graded as having no, mild, moderate, or severe hepatic steatosis. Of the 13,983 participants with hepatic imaging records, 13,856 of them could be graded [7]. Detailed descriptions and procedures have been provided previously [7,11]. In the absence of a standard definition, we defined NAFLD as moderate or severe hepatic steatosis, and non-NAFLD as no or mild hepatic steatosis [12]. The overall intra-rater and inter-rater κ statistics for reliability of the dichotomized outcomes (“no or mild” and “moderate or severe”) were 0.77 (95% CI 0.73 to 0.82) and 0.70 (95% CI 0.64 to 0.76), respectively [11].

### Non-invasive markers of NAFLD

The non-invasive markers of NAFLD were calculated based on the equations reported in the literature [6–9]. In brief, FLI includes body mass index (BMI), γ-glutamyltranspeptidase, triglycerides, and waist circumference; HSI includes aspartate aminotransferase (AST)/alanine aminotransferase (ALT) ratio, BMI, diabetes, and sex; LAP includes sex, triglycerides, and waist circumference; and LFS includes AST/ALT ratio, diabetes, fasting AST level, fasting insulin level, and MetS. The SteatoTest was not included in the current study, as this test is a commercially one, and the calculation formula is not disclosed. The threshold used in the current study also adopted the cutoff points suggested in the literature: the high/low cutoff points were ≥1.257/≤ −1.413, ≥30/<30, and ≥30/<30 for LFS, HSI, and FLI, respectively [6,7,9].

### Mortality follow-up

In NHANES III, cause of death was coded using the International Classification of Diseases. 10th Revision (ICD-10). ICD codes I00 to I78 and E10 to E14 were used to assess cardiovascular and diabetes mortality, respectively, as in our previous studies [13,14]. Malignancy and liver mortality were defined by the Underlying Cause of Death (UCOD)_113 20 to 23, 25 to 26, and 43, and UCOD_113 15, 24, and 93 to 95, respectively, as in the literature [15]. The length of follow-up was the time from the study examination date to death or to December 31, 2006, whichever was earlier.

### Definition of diabetes, hypertension, and MetS

Diabetes was defined according to the latest American Diabetes Association (ADA) guideline, which includes fasting glucose ≥126 mg/dl, random plasma glucose ≥200 mg/dl, or A1C ≥ 6.5. Patients were considered to have hypertension if they had systolic blood pressure (SBP) ≥140 or diastolic blood pressure (DBP) ≥90 mmHg, or if they were receiving anti-hypertensive drug therapy. MetS was defined according to the joint scientific statement on harmonizing MetS [16], that is, having three or more of the following factors: 1) elevated blood pressure (SBP ≥ 130 mmHg and/or DBP ≥85 mmHg and/or being in receipt of anti-hypertension drug therapy); 2) elevated triglycerides (≥150 mg/dl (1.7 mmol/l) and/or being in receipt of drug treatment for elevated triglycerides); 3) reduced high-density liproprotein (HDL) cholesterol (<40 mg/dl (1.0 mmol/l) in men and <50 mg/dl (1.3 mmol/l) in women and/or being in receipt of drug treatment for elevated HDL cholesterol); 4) elevated fasting glucose (≥100 mg/dl (5.6 mmol/l) and/or being in receipt of treatment for elevated glucose); and 5) large waist circumference (>102 cm in men and >88 cm in women of European descent). Liver fat percentage was estimated using the equation reported in the same literature as the LFS [7]. The equation includes the same variables as the LFS, but with a different calculation.

### Statistical analysis

To assess model discrimination, we calculated the area under curve (AUC) for the receiver operating characteristic (ROC) for each non-invasive score of NAFLD. The difference between two AUCs was compared using the maximum likelihood estimation method [17] and implemented using ROCKIT [18]. Sensitivity, specificity, positive likelihood ratio (+LR), negative likelihood ratio (−LR), and corresponding 95% CIs were also calculated. The non-invasive NAFLD measurement with the best performance (in terms of AUC for ROC) was selected and evaluated for its association with mortality.

In the Cox proportional hazard regression model, non-invasive score was modeled as threshold and continuous variables. Using the lower threshold as the reference, the hazard ratio (HR) and 95% CI for the highest threshold were calculated using the simple and fully adjusted Cox regression models. In the simple model, we adjusted for age and sex. In the full model, we adopted the adjustment model of a recent study related to NAFLD fibrosis and mortality [15], which includes age, sex, race/ethnicity, income, education, diabetes, hypertension, use of lipid-lowering medication, smoking, drinking, history of cardiovascular disease (CVD), waist circumference, dietary caffeine intake, HDL cholesterol, triglycerides, transferrin saturation and C-reactive protein. *P* ≤ 0.05 was considered significant. The proportional hazards assumption was evaluated by including time-dependent covariates in the full regression model; the overall test of proportional hazards was not significant (*P* > 0.05) suggesting that the proportional assumption was valid. To gain additional insight into the potential nonlinearity of the effect of LFS, we examined the Cox regression models using penalized spline. Two degrees of freedom (df) used in the spline because the model had the lowest Akaike’s information criterion (AIC) (best fit) when df = 2. Sample weights that accounted for the unequal probabilities of selection, oversampling, and non-response were applied in all analyses using the complex sampling module in SPSS (V18.0; SPSS Inc, Chicago, IL, USA) or R software (V2.15.0) [19]. All values presented were weighted to represent the civilian population of the USA.

We also evaluated the ability in risk reclassification using integrated discrimination improvement (IDI) [20] and category-less net reclassification improvement (NRI) [20]. IDI was used to compare the difference in discrimination slopes [21], while category-less NRI was used to compare classifications from two models for changes by outcome for a net calculation of changes in the right direction. Estimated risk of death of different models was calculated using the equation of 1/(1 + exp(−1 × XBeta)). Analyses were performed using R software (V2.15.0) [19].

## Results

### Cross-sectional NAFLD prediction cohort

Of the 5,184 participants included for the AUC study, 18.4% (16.5 to 20.4%) had NAFLD. The characteristics of this cohort are provided in Table 1. For NAFLD prediction, LFS was the best performer for predicting NAFLD, with an AUC of 0.771 (*P* < 0.001), whereas the lowest AUC (0.732) was observed for HSI (Table 2). Using maximum likelihood estimation, the difference between the AUC of LFS and other markers (FLI, LAP, and HSI) was statistically significant (all *P* < 0.01). Interestingly, the diagnostic accuracy of these markers differed by race/ethnicity (Table 2). The sensitivity and specificity, and the + LR and − LR of the suggested high and low cutoff points for excluding/including NAFLD are provided in Table 3 and Additional file 1: Table S1, respectively. The raw number used to calculate the diagnostic accuracy and the characteristics of true and false positive, and true and false negative (based on the LFS threshold) are provided in Additional file 1: Tables S2 and S3.

**Table 1:** **Characteristics of participants in the cross-sectional NAFLD prediction cohort according to NAFLD status** ^**a**^

**Characteristics**	**No NAFLD (n = 4,117)**		**NAFLD (n = 1,067)**		***P*** **value**
	**Value**	**95% CI**	**Value**	**95% CI**	
Age, years	40.42	39.59 to 41.25	45.32	43.97 to 46.67	<0.001
Female sex, %	52.6	50.4 to 54.7	45.6	41.7 to 49.5	0.005
Race/ethnicity, %					<0.001
Non-Hispanic white	74.9	71.2 to 78.3	73.6	69.6 to 77.3	
Non-Hispanic black	11.6	10.1 to 13.4	8.7	7.3 to 10.3	
Mexican–American	4.7	3.7 to 5.8	7.6	6.2 to 9.3	
Other	8.8	6.5 to 11.8	10.1	7.3 to 13.8	
Education					0.001
< High school	5.5	4.2 to 7.1	8.4	6.4 to 11.1	
High school	14.1	12.5 to 16.0	18.9	15.6 to 22.8	
> High school	80.4	77.7 to 82.8	72.6	67.8 to 77.0	
Smoking					<0.001
Never	48.6	46.2 to 51.1	41.9	38.1 to 45.7	
Former	23.2	21.5 to 25.1	32.8	29.4 to 36.5	
Current	28.1	25.8 to 30.6	25.3	21.7 to 29.3	
Diabetes, %	1.9	1.6 to 2.2	9.8	7.6 to 12.5	<0.001
Hypertension, %	16.0	14.6 to 17.6	33.7	29.9 to 37.6	<0.001
Lipid-lowering medication, %	2.4	1.8 to 3.0	5.0	3.4 to 7.1	<0.001
History of CVD, %	1.8	1.3 to 2.3	3.9	2.8 to 5.4	0.002
Metabolic syndrome, %	16.9	15.3 to 18.7	54.8	51.2 to 58.5	<0.001
Abdominal obesity, %	28.0	26.5 to 29.5	65.2	61.3 to 68.9	<0.001
Hypertriglyceridemia, %	22.8	20.5 to 25.3	54.3	50.4 to 58.2	<0.001
Impaired fasting glucose, %	20.1	18.5 to 21.8	42.1	38.4 to 46.0	<0.001
Low HDL level, %	35.50	32.7 to 38.5	60.1	54.9 to 65.0	<0.001
Body mass index, kg/m^2^	25.72	25.51 to 25.93	30.29	29.76 to 30.82	<0.001
Waist circumference, cm	88.82	88.35 to 89.3	101.75	100.36 to 103.13	<0.001
Serum cholesterol, mg/dl	199.83	198.5 to 201.17	208.63	205.63 to 211.63	<0.001
Serum triglycerides, mg/dl	117.71	114.18 to 121.24	192.30	183.44 to 201.15	<0.001
Serum HDL cholesterol, mg/dl	51.04^b^	50.15 to 51.93	42.91^d^	41.67 to 44.15	<0.001
Plasma glucose, mg/dl	94.07^c^	93.41 to 94.73	104.95	102.56 to 107.33	<0.001
Serum insulin, μU/ml	8.80	8.54 to 9.06	16.09	14.85 to 17.33	<0.001
SBP, mmHg	118.42	117.55 to 119.3	125.7^e^	124.7 to 126.7	<0.001
AST, U/L	19.60	19.21 to 20	25.24	24.31 to 26.17	<0.001
ALT, U/L	15.70	15.03 to 16.36	25.68	24.27 to 27.09	<0.001
GGT, U/L	24.42	23.69 to 25.15	41.61	37.77 to 45.44	<0.001
FLI	34.70	33.41 to 35.99	67.56	64.92 to 70.2	<0.001
HSI	33.10	32.7 to 33.49	39.26	38.64 to 39.89	<0.001
LFS	−1.92	−2.03 to to 1.82	0.21	−0.04 to 0.46	<0.001
Liver fat percentage, %	2.45	2.33 to 2.56	5.88	5.49 to 6.27	<0.001
LAP	39.74	38.08 to 41.40	91.71	86.35 to 97.06	<0.001

ALT, alanine aminotransferase: AST, aspartate aminotransferase; CVD, cardiovascular disease; FLI, fatty liver index; GGT, gamma-glutamyl transferase; HDL, high-density lipoprotein; HSI, hepatic steatosis index; LAP, lipid accumulation product; LFS, liver fat score; NAFLD, non-alcoholic fatty liver disease SBP, systolic blood pressure.

^a^No NAFLD: no or mild hepatic steatosis; NAFLD: moderate or severe hepatic steatosis.

Numbers of participants: ^b^4108; ^c^4109; ^d^1061; ^e^1049.

**Table 2:** **Quality of prediction scores in predicting NAFLD**

**NAFLD prediction scores**	**All participants (n = 5,184; 1067 cases)**		**Non-Hispanic white (n = 1,953; 376 cases)**		**Non-Hispanic black (n = 1,577; 244 cases)**		**Mexican-American (n = 1,409; 401 cases)**		**Others (n = 245; 46 cases)**	
	**AUC**	**95% CI**	**AUC**	**95% CI**	**AUC**	**95% CI**	**AUC**	**95% CI**	**AUC**	**95% CI**
LFS	0.771	0.754 to 0.787	0.78	0.753 to 0.808	0.711	0.674 to 0.748	0.781	0.754 to 0.808	0.865	0.804 to 0.926
FLI	0.757	0.74 to 0.774	0.778	0.75 to 0.807	0.706	0.668 to 0.745	0.764	0.737 to 0.791	0.788	0.708 to 0.867
LAP	0.741	0.723 to 0.758	0.767	0.739 to 0.795	0.694	0.654 to 0.733	0.726	0.698 to 0.755	0.761	0.683 to 0.84
HSI	0.732	0.714 to 0.749	0.735	0.705 to 0.764	0.673	0.635 to 0.711	0.760	0.733 to 0.787	0.779	0.705 to 0.854

AUC, area under the curve; FLI, fatty liver index; HSI, hepatic steatosis index; LAP, lipid accumulation product; LFS, liver fat score; NAFLD, non-alcoholic fatty liver disease.

All *P* < 0.001.

**Table 3:** **Sensitivity and specificity of exclusion/inclusion cutoff points of LFS, FLI, and HSI in the cross-sectional NAFLD prediction cohort and literature**

**NAFLD definition**	**Non-invasive NAFLD score**	**Cutoff point**		**SP, %**	**95% CI, %**	**SN, %**	**95% CI, %**	**Reported SP, %**	**Reported SN, %**	**Ref**
No to mild versus intermediate to severe steatosis)	LFS	Inclusion	≥1.257	96.43	95.82 to 96.98	26.34	23.71 to 29.09	95	51	[7]
		Exclusion	≤ − 1.413	67.94	66.49 to 69.36	73.95	71.20 to 76.56	52	95	
	FLI	Inclusion	≥60	73.60	72.22 to 74.94	67.48	64.58 to 70.29	86	61	[6]
		Exclusion	<30	48.77	47.24 to 50.31	84.44	82.13 to 86.57	64	87	
	HSI	Inclusion	≥36	69.35	67.91 to 70.75	66.26	63.33 to 69.10	92	46	[9]
		Exclusion	<30	29.75	28.36 to 31.18	91.38	89.53 to 92.99	40	92.50	

AUC, area under the curve; FLI, fatty liver index; HSI, hepatic steatosis index; LAP, lipid accumulation product; LFS, liver fat score; NAFLD, non-alcoholic fatty liver disease; SN, sensitivity; SP, specificity.

### Prospective mortality prediction cohort

Of the several NAFLD non-invasive prediction scores tested, the LFS gave the best performance. As our second aim was to investigate the relationship of the best non-invasive score with outcome, we tested if LFS was associated with mortality. Table 4 shows the characteristics of the participants. During a median follow-up of 14.7 years (range 0.1 to 18.2 years) and 83,830.5 person-years, 793, 311, 209, 58, and 17 participants died from all, cardiovascular-, malignancy-, diabetes-, and liver-related causes, respectively. Higher LFS were associated with all causes of mortality tested, except malignancy-related causes.

**Table 4:** **Characteristics of participants in the prospective mortality prediction cohort according to different LFS thresholds**

**Characteristics**	**Low LFS (n = 3,524)**		**Intermediate LFS (n = 1,890)**		**High LFS (n = 478)**		***P*** **value**
	**Value**	**95% CI**	**Value**	**95% CI**	**Value**	**95% CI**	
Age, years	39.23	38.34 to 40.13	45.50	44.28 to 46.71	45.04	42.78 to 47.29	<0.001
Sex, female %	56.40	54.1 to 58.7	42.30	39.3 to 45.3	45.70	38.1 to 53.5	<0.001
Race/ethnicity, %							<0.001
Non-Hispanic white	78.40	75.5 to 81.1	74.10	69.3 to 78.4	73.30	68.0 to 78.0	<0.001
Non-Hispanic black	10.30	8.9 to 11.8	10.50	8.8 to 12.5	10.90	8.6 to 13.7	<0.001
Mexican–American	4.20	3.5 to 5.1	6.40	5.1 to 8.0	7.80	5.3 to 11.2	<0.001
Other	7.10	5.4 to 9.3	9.00	5.9 to 13.4	8.00	5.6 to 11.5	<0.001
Education, %							<0.001
< High school	4.10	3.2 to 5.2	8.20	6.0 to 11.0	8.10	5.5 to 11.7	<0.001
High school	13.30	11.7 to 15.0	18.80	15.6 to 22.4	16.80	13.8 to 20.3	<0.001
> High school	82.60	80.3 to 84.7	73.10	68.4 to 77.3	75.10	70.1 to 79.6	<0.001
Smoking, %							<0.001
Never	49.20	46.2 to 52.3	41.00	37.3 to 44.8	47.90	40.3 to 55.5	<0.001
Former	21.60	19.6 to 23.7	33.20	29.5 to 37.1	32.30	25.9 to 39.5	<0.001
Current	29.20	26.4 to 32.2	25.80	23.1 to 28.6	19.80	14.1 to 27.1	<0.001
Diabetes, %	0.20	0.1 to 0.3	5.70	4.6 to 7.0	26.20	21.3 to 31.7	<0.001
Hypertension, %	10.00	8.7 to 11.4	32.70	29.6 to 36.0	45.40	39.6 to 51.3	<0.001
Lipid-lowering medication, %	1.10	.7 to 1.8	5.40	4.1 to 7.1	7.20	4.4 to 11.6	<0.001
History of CVD, %	1.30	.9 to 1.9	3.40	2.4 to 4.8	5.10	3.1 to 8.2	<0.001
Poverty income ratio	3.25	3.09 to 3.4	2.98	2.76 to 3.2	2.83	2.54 to 3.11	0.003
Waist circumference, cm	85.22	84.74 to 85.71	100.22	99.52 to 100.92	112.22	110.51 to 113.93	<0.001
Dietary caffeine intake, mg	272.81	251.75 to 293.87	276.43	234.41 to 318.45	216.06	189.12 to 243	0.222
Serum cholesterol, mg/dl	196.06	194.01 to 198.11	213.02	209.91 to 216.14	218.33	211.56 to 225.1	<0.001
Serum HDL cholesterol, mg/dl	53.38	52.51 to 54.25	43.81	42.74 to 44.89	39.06	37.89 to 40.23	<0.001
Serum transferrin saturation, %	27.78	27.25 to 28.31	26.25	25.55 to 26.95	25.54	23.88 to 27.2	<0.001
Serum C-reactive protein, mg/dl	0.33	0.31 to 0.35	0.45	0.42 to 0.49	0.58	0.5 to 0.66	<0.001
Drinks/week^a^	2.68	2.45 to 2.9	2.32	2.01 to 2.64	1.62	1.11 to 2.13	<0.001
LFS	−2.53	−2.58 to −2.48	−0.41	−0.46 to −0.36	3.20	2.83 to 3.57	<0.001
Mortality							
All causes	8.90	7.7 to 10.2	13.60	11.4 to 16.2	17.70	12.9 to 23.9	<0.001
CVD-related	2.80	2.3 to 3.5	5.10	4.0 to 6.4	9.00	5.4 to 14.8	<0.001
Liver-related	0.10	0.0 to 0.3	0.10	0.0 to 0.2	1.20	0.3 to 5.2	0.069
Diabetes-related	0.20	0.1 to 0.5	0.90	0.6 to 1.5	3.90	2.0 to 7.4	<0.001
Malignancy-related	2.80	2.1 to 3.8	3.60	2.5 to 5.2	2.00	1.0 to 4.2	0.698

CVD, cardiovascular disease; LFS, liver fat score; NAFLD, non-alcoholic fatty liver disease.

^a^A drink was defined as a 12-oz beer, a 4-oz glass of wine, or 1-oz of liquor (spirits).

**Figure 1:**
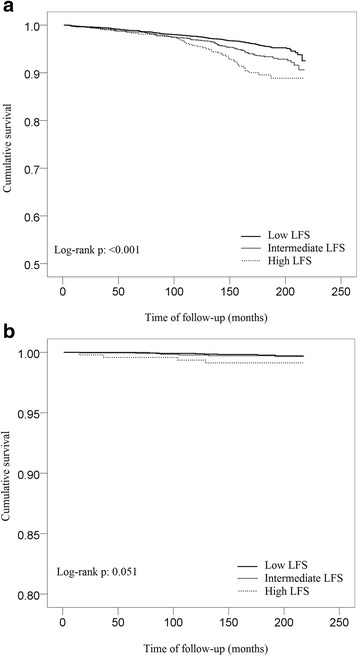
**Kaplan–Meier curves of mortality curves according to different liver fat score (LFS) thresholds. (a)** Cardiovascular-related and **(b)** liver-related mortality.

**Figure 2:**
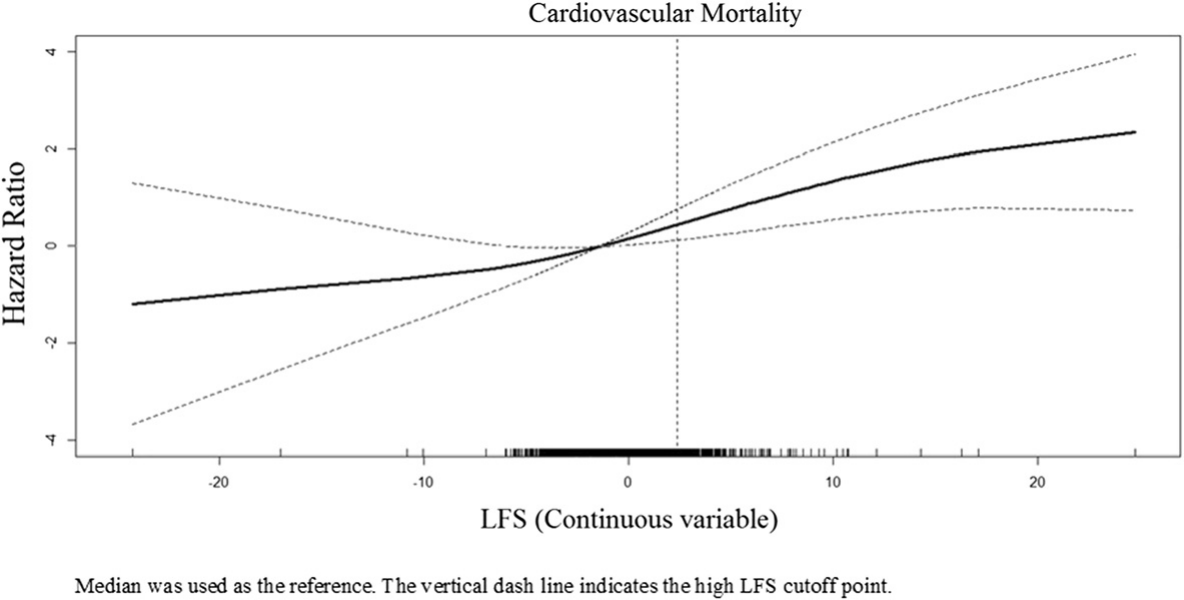
**Association between liver fat score (LFS) and cardiovascular mortality via penalized regression splines.**

The results of Cox regression analysis are shown in Table 5. Participants in the high LFS group had a 60% higher risk (HR = 1.6; 95% CI 1.01 to 2.54; *P* = 0.048 in full model) of all-cause mortality than the intermediate LFS group. For cardiovascular mortality, participants in the high LFS group was associated with 2.24-fold (95% CI 1.03 to 4.88; *P* = 0.042 in full model) and 2.3-fold (95% CI 1.19 to 4.48; *P* = 0.015 in full model) increase in risk of death compared with the low and intermediate LFS groups. For liver mortality, participants in the high LFS group had a 31.25-fold (95% CI 3.13 to 333.33; *P* = 0.004 in full model) and 30.3-fold (95% CI 4 to 250; *P* = 0.001 in full model) increase in risk of death compared with the low and intermediate LFS groups. The Kaplan–Meier survival curves for cardiovascular and liver-related mortality are provided in Figure 1. When LFS was treated as continuous variable, a one-unit increase of LFS was associated with increased mortality of all-cause and cardiovascular-, liver-, and diabetes-related mortality, with HRs of 1.09 (95% CI 1.01 to 1.19; *P* = 0.039), 1.11 (95% CI 1.03 to 1.19; *P* = 0.006), 1.32 (95% CI 1.12 to 1.55; *P* = 0.001), and 1.21 (95% CI 1.02 to 1.44; *P* = 0.034), respectively (Table 5), after full adjustment. Similar results were obtained after further adjustment of the NAFLD fibrosis score (NFS). The relationship between LFS and cardiovascular mortality as examined by penalized regression spline is shown in Figure 2.

**Table 5:** **Association between LFS and mortality**

**Mortality from:**	**Simple model** ^**a**^			**Full model** ^**b**^			**Full model + NFS adjustment** ^**b**^		
	**HR**	**95% CI**	***P*** **value**	**HR**	**95% CI**	***P*** **value**	**HR**	**95% CI**	***P*** **value**
All causes									
Low (ref) versus high LFS	1.51	0.99 to 2.3	0.056	1.59	0.93 to 2.72	0.087	1.65	0.96 to 2.85	0.07
Int (ref) versus high LFS	1.52	1.03 to 2.23	0.034	1.60	1.01 to 2.54	0.048	1.61	1 to 2.58	0.049
LFS (continuous)	1.07	0.99 to 1.14	0.082	1.09	1.01 to 1.19	0.039	1.1	1.024 to 1.19	0.011
CVD									
Low (ref) versus high LFS	2.29	1.24 to 4.26	0.009	2.24	1.03 to 4.88	0.042	2.30	1.02 to 5.19	0.046
Int (ref) versus high LFS	2.20	1.18 to 4.12	0.015	2.30	1.19 to 4.48	0.015	2.32	1.18 to 4.55	0.015
LFS (continuous)	1.11	1.04 to 1.19	0.002	1.11	1.03 to 1.19	0.006	1.13	1.05 to 1.21	<0.001
Liver disease									
Low (ref) versus high LFS	9.80	2.01 to 47.62	0.006	31.25	3.13 to 333.33	0.004	46.45	5.13 to 420.51	<0.001
Int (ref) versus high LFS	17.24	2.6 to 111.11	0.004	30.30	4–250	0.001	32.99	4.57 to 237.95	<0.001
LFS (continuous)	1.25	1.14 to 1.36	<0.001	1.32	1.12 to 1.55	0.001	1.3	1.12 to 1.51	<0.001
Diabetes									
Low (ref) versus high LFS	15.87	6.02 to 41.67	<0.001	2.87	0.65 to 12.66	0.161	3.13	0.67 to 14.56	0.143
Int (ref) versus high LFS	5.24	2.24 to 12.2	<0.001	1.98	0.71 to 5.52	0.185	2.04	0.72 to 5.79	0.176
LFS (continuous)	1.37	1.27 to 1.48	<0.001	1.21	1.02 to 1.44	0.034	1.21	1.03 to 1.42	0.024
Malignancy									
Low (ref) versus high LFS	0.55	0.25 to 1.24	0.147	0.75	0.27 to 2.11	0.578	0.75	0.27 to 2.09	0.576
Int (ref) versus high LFS	0.65	0.29 to 1.44	0.281	0.76	0.32 to 1.79	0.519	0.76	0.32 to 1.79	0.518
LFS (continuous)	0.92	0.86 to 0.99	0.026	0.97	0.87 to 1.07	0.521	0.95	0.8 to 1.12	0.495

CVD, cardiovascular disease; Int, intermediate; LFS, liver fat score.

^a^Simple model: adjusted for age and sex.

^b^Full model: further adjusted for race/ethnicity, education, income, diabetes, hypertension, history of CVD, lipid-lowering medication, smoking status, waist circumference, alcohol consumption, caffeine consumption, total cholesterol, high-density lipoprotein cholesterol, transferrin saturation, and C-reactive protein; Because of smaller numbers of liver- and diabetes-related deaths, we did not adjust for education, income, history of CVD, lipid-lowering medication, and C-reactive protein in the full model.

Age, sex, hypertension, and diabetes are commonly used in assessing risk of mortality. We therefore evaluated whether addition of LFS (categorical: low/intermediate/high risk of NAFLD) in a basic clinical model composed of these traditional risk factors could improve the risk prediction. Risk reclassification with IDI showed a modest positive shift to improvement when LFS was added in the basic clinical model (IDI: 0.0131; 95% CI 0.009 to 0.017; *P* < 0.001). Similar result was observed using category-less NRI (NRI: 0.133; 95% CI 0.054 to 0.211; *P* < 0.001).

## Discussion

Using a large, nationally representative cohort with more than 10 years of follow-up and ultrasonographic data, we have demonstrated that LFS is the best prediction score for ultrasonography-diagnosed NAFLD, and can predict mortality, including cardiovascular- and liver-related mortality.

It is important to find an easy and cost-effective way to screen for NAFLD. Of the several non-invasive scores we tested, LFS showed the best performance in identifying ultrasonography-diagnosed NAFLD. Notably, LFS was derived using ^1^H-MRS-diagnosed NAFLD, whereas FLI, HSI, and LAP were derived from ultrasonography-diagnosed NAFLD. For those scores derived from ultrasonography, the definitions of the NAFLD were also somewhat different (see Additional file 1: Table S4). Ultrasonography is a semi-quantitative imaging technique, and the definitions of NAFLD differed between studies. By contrast, ^1^H-MRS is by far the most sensitive and quantitative imaging tool in identifying hepatic steatosis. This could be the reason why the non-invasive score (LFS) derived from ^1^H-MRS performed better and more robustly in identifying cases in the current study. As there is no standard definition of ultrasonography-diagnosed NAFLD for good measurement, we used three additional definitions to test the performance of different non-invasive indices (see Additional file 1: Table S5), and the LFS still came out best. We evaluated whether combining all prediction scores (combined score) could improve the NAFLD prediction. The AUC of the combined score increased to 0.782 (95% CI 0.766 to 0.798), suggesting that there are unique NAFLD predicting components being captured in different prediction scores.

Interestingly, there was a difference in the diagnostic accuracy of the different non-invasive scores, with the lowest diagnostic accuracy being observed in black patients for all tested scores, suggesting that the clinical risk factors of NAFLD could be ethnicity-specific and particularly different in black populations. Like other disease predictions [22], deriving an ethnicity- or population-specific prediction model may be required to achieve a high accuracy of NAFLD prediction. Notably, although there was an observed difference in the diagnostic accuracy of LFS for NAFLD, no significant interaction (*P* > 0.05) between LFS and race/ethnicity on mortality was observed, therefore, no subgroup analysis was performed in the subsequent analyses.

LFS was calculated based on the AST/ALT ratio, diabetes, fasting AST level, fasting insulin level, and MetS. Given that diabetes and MetS are known to be associated with mortality, the association between LFS and mortality could be attributable to these factors. However, the components of MetS were adjusted for in the full model, suggesting that the association of LFS with mortality may be independent of these factors. A number of different organizations use different recommended waist circumference thresholds for abdominal obesity in defining MetS. In addition to the threshold suggested by ATPIII [16], we also used the population-specific threshold suggested by the International Diabetes Federation, and the findings remained unchanged (data not shown).

In the literature on FLI, LFS, and HSI, various high and low cutoff points have been proposed to include and exclude NAFLD [6,7,9]. The high cutoff point should have a high specificity and + LR, while the low cutoff point should have a high sensitivity and low − LR. In general, the diagnostic performance of the defined cutoff points of the NAFLD prediction scores was not the same as that originally reported in the literature because the sample populations were different (Table 3). The high cutoff point of LFS had a slightly higher specificity (96.4%) in the current study than the figure (95%) reported in the literature, meaning that study participants with a high LFS were very likely to have a higher risk of mortality.

Two previous studies validated the non-invasive prediction scores in adults [23,24]. Koehler *et al*. validated FLI and LAP in 2,652 participants in the Rotterdam Study. FLI and LAP had an AUC of 0.813 and 0.786, respectively, in predicting ultrasonography-diagnosed NAFLD. FLI had a higher AUC in the Rotterdam Study than in the current study. Interestingly, the Rotterdam Study used the scoring protocol of Hamaguchi *et al*. [25], and the one used by NHANES was an algorithm derived based on that same publication. The Rotterdam Study was a population-based cohort study of elderly inhabitants of a district of Rotterdam, whereas NHANES was a nationally representative population-based study with participants of different races/ethnicities and age. LFS was previously validated in a study of 40 non-diabetic patients with biopsy-proven NAFLD and 85 healthy controls [23], which showed that LFS had an AUC of 0.86. Although the AUC from different validation studies cannot be compared directly, our study is in agreement with previous validation studies showing that LFS had the highest AUC, followed by FLI and LAP. Although no validation study has been performed for HSI, our study suggested that HSI is better than LAP as a predictor of NAFLD.

Although identifying people with NAFLD is important, identifying people with adverse clinical outcome is even more important, as NAFLD consists of a wide spectrum of conditions, ranging from simple steatosis to cirrhosis with varying prognosis. In concordance with previous NHANES reports [12,15], our study did not reveal any significant association between ultrasonography-diagnosed NAFLD and mortality (data not shown). This finding is intriguing. Ultrasonography-diagnosed NAFLD is not associated with mortality, whereas LFS, a marker of NAFLD, is associated with mortality. This could be due to the reason mentioned earlier, namely, that LFS was derived using the sensitive and quantitative imaging tool ^1^H-MRS, whereas other NAFLD prediction scores were derived using a less sensitive semi-quantitative ultrasonography. In fact, we found no association of other markers of NAFLD with mortality (se Additional file 1: Table S5), further suggesting that ^1^H-MRS-derived LFS may be more superior in identifying NAFLD and predicting clinical outcome.

We then investigated which individual component of the LFS was associated with CVD mortality in the multivariable model, and found the only significant association observed Qa with fasting serum insulin (estimate of 1.02; 95% CI 1.01 to 1.03; *P* < 0.001), suggesting that fasting serum insulin may be the main driver for the observed association.

Notably, high LFS is also associated with low transferrin saturation (Table 4). In our previous study, we showed that low transferrin saturation was robustly associated with pre-diabetes [26]. These observations suggested that elevated insulin resistance might be the key factor leading to mortality in people with NAFLD, which may also explain why LFS can predict mortality whereas ultrasonography-diagnosed NAFLD cannot. Another possibility is that high LFS indicates the presence of other NAFLD-related conditions, such as NASH and fibrosis. In participants with NAFLD, high LFS is associated with high NFS (data not shown), which is a prediction score of NAFLD fibrosis [27], although NFS does not predict NAFLD (data not shown) nor is it significantly associated with mortality in the general population (see Additional file 1: Table S5). However, further adjustment of NFS revealed that the effect of LFS is independent of NFS (Table 5), suggesting that the association between LFS and mortality may be independent of NAFLD fibrosis. Future study is required to confirm our observations and to examine the underlying mechanisms.

Age, sex, and presence of diabetes or hypertension are simple risk factors that are commonly used by clinicians to evaluate mortality risk. We showed that LFS has an independent role in predicting mortality and improved risk reclassification. Similarly, although the Framingham Risk Score (FRS) was not intended for use in mortality prediction, we found that the associations of LFS with cardiometabolic disease related mortality were independent of FRS (see Additional file 1: Table S6). These findings suggest that abnormal liver function may play a role in mortality determination, independently of traditional risk factors.

Our study has several strengths. The study population is large, multiethnic, nationally representative, and well-characterized, with data on ultrasonography-diagnosed NAFLD, multiple risk factors, and potential confounders. The long follow-up and the large number of events provided ample statistical power. The wide range of collected data from NHANES III allowed construction of four different non-invasive prediction scores simultaneously, so that they could be compared in parallel and with different definitions of NAFLD.

Nevertheless, there are limitations. The major limitation of the current study is the use of ultrasonography-diagnosed NAFLD, which can lead to misclassification error. In the absence of a standard definition, we defined NAFLD as presence of moderate or severe hepatic steatosis, while non-NAFLD was defined as presence of no or mild hepatic steatosis, as in previous study. The case definition, especially when mild hepatic steatosis was defined as non-NAFLD, could be a potential source of bias. This classification could have led to underestimation of the NAFLD prevalence in the current study, which was reported to be 20% to 33% in the general population [28], although the lower prevalence observed could also be due to the lower prevalence of obesity in the current study [29]. It is acknowledged that ultrasonography has limited sensitivity and specificity in diagnosing NAFLD, especially when less than 33% of the liver parenchyma is infiltrated by fat [30,31] and in the presence of liver cirrhosis that may lead to decreased hepatic steatosis. To confirm our findings, we defined NAFLD in different ways and still found that LFS was the best marker of NAFLD (Table 4; see Additional file 1: Table S7), and participants with mild hepatic steatosis also did not have increased mortality compared with those without hepatic steatosis (see Additional file 1: Table S8).

Although liver biopsy is considered the gold standard in diagnosing NAFLD, it is not justifiable to perform liver biopsy in large numbers of asymptomatic individuals, therefore ultrasonography is still considered an acceptable first-line screening procedure for NAFLD in clinical practice [32]. However, ultrasonography cannot distinguish between NASH, fibrosis or cirrhosis.

The prediction score named the SteatoTest, was not included in the current study; whether it is superior to LFS or otherwise requires further study.

In the Cox regression analysis, there were too few cases of liver-related mortality, which led to unreliable estimates, and could also be a potential source of bias, therefore cautious interpretation is required.

## Conclusion

In conclusion, we found that ^1^H-MRS derived NAFLD prediction score LFS was the most robust non-invasive score identifying NAFLD in this US population and predicted mortality. NAFLD is highly prevalent, and can be associated with morbidity and mortality if left unidentified. Our findings suggest that LFS may be a promising tool for large-scale NAFLD screening. If confirmed in future studies, LFS may be a useful marker for large-scale NAFLD screening and prediction of long-term clinical outcomes.

## Additional file

Additional file 1:
**Additional file contains supplementary Tables S1 to S8 and supplementary Figure S1.**
