# Supplementary material for: Non-invasive score identifies ultrasonography-diagnosed non-alcoholic fatty liver disease and predicts mortality in the USA
Source: BMC Med. 2014 Sep 10;12:154. doi: 10.1186/s12916-014-0154-x (PMC4207316; doi:10.1186/s12916-014-0154-x)

Supplementary Table 1. Positive likelihood ratio, negative likelihood ratio, positive predictive value, and negative predictive value of ruling in and out cutoff points of LFS, FLI, and HSI in the cross-sectional NAFLD prediction cohort.

| NAFLD definition | Non-invasive NAFLD score | Cutoff | Cutoff Point | +LR | 95% CI | -LR | 95% CI | PPV | 95% CI | NPV | 95% CI |
| --- | --- | --- | --- | --- | --- | --- | --- | --- | --- | --- | --- |
| NAFLD (none to mild vs. intermediate to severe steatosis) | LFS | Ruling in | ≥1.257 | 7.38 | (6.11 - 8.90) | 0.76 | (0.74 - 0.79) | 65.65 | (60.94-70.15) | 83.47 | (82.39-84.52) |
|  |  | Ruling out | ≤-1.413 | 2.31 | (2.18 - 2.44) | 0.38 | (0.35 - 0.43) | 37.41 | (35.34-39.52) | 90.96 | (89.89-91.95) |
|  | FLI | Ruling in | ≥60 | 2.56 | (2.39 - 2.73) | 0.44 | (0.40 - 0.48) | 39.85 | (37.58-42.15) | 89.72 | (88.65-90.73) |
|  |  | Ruling out | <30 | 1.65 | (1.58 - 1.71) | 0.32 | (0.28 - 0.37) | 29.93 | (28.30-31.61) | 92.36 | (91.17-93.45) |
|  | HSI | Ruling in | ≥36 | 2.16 | (2.03 - 2.30) | 0.49 | (0.45 - 0.53) | 35.91 | (33.78-38.07) | 88.8 | (87.66-89.87) |
|  |  | Ruling out | <30 | 1.3 | (1.27 - 1.34) | 0.29 | (0.24 - 0.35) | 25.21 | (23.85-26.61) | 93.01 | (91.50-94.33) |
| +LR: positive likelihood ratio; -LR: negative likelihood ratio; PPV: positive predictive value; NPV: negative predictive value | | | | | | | | | | | |

Supplementary Tables 2. The raw number that used to calculate the diagnostic accuracy of NAFLD.

| NAFLD | LFS score | | | FLI score | | | HSI score | | |
| --- | --- | --- | --- | --- | --- | --- | --- | --- | --- |
|  | Low | Intermediate | High | Low | Intermediate | High | Low | Intermediate | High |
| None + mild | 2797 | 1173 | 147 | 2008 | 1022 | 1087 | 1225 | 1630 | 1262 |
| Moderate + Severe | 278 | 508 | 281 | 166 | 181 | 720 | 92 | 268 | 707 |

Supplementary Table 3. The characteristics of true and false positive, and true and false negative (based on the LFS threshold).

| With NAFLD |  | False Negative | | | |  | True Positive | |
| --- | --- | --- | --- | --- | --- | --- | --- | --- |
| Characteristics |  | Low LFS (N=278) | | Intermediate LFS (N=508) | |  | High LFS (N=281) | |
| AST/ALT ratio |  | 1.53 | (1.46-1.6) | 1.08 | (1.02-1.15) |  | 0.95 | (0.89-1.01) |
| Aspartate aminotransferase: SI(U/L) |  | 18.97 | (18.02-19.92) | 22.88 | (21.95-23.82) |  | 36.31 | (33.01-39.61) |
| Alanine aminotransferase: SI (U/L) |  | 13.84 | (12.81-14.87) | 24.12 | (22.31-25.93) |  | 41.11 | (36.99-45.24) |
| Serum insulin (uU/mL) |  | 7.79 | (7.35-8.23) | 13.19 | (12.73-13.66) |  | 30.33 | (26.94-33.72) |
| Abdominal_obesity, % |  | 25.7% | (19.8%-32.7%) | 72.8% | (66.9%-77.9%) |  | 92.5% | (85.6%-96.2%) |
| Hypertrigeridemia, % |  | 17.7% | (12.6%-24.2%) | 60.4% | (55.0%-65.6%) |  | 81.4% | (73.8%-87.2%) |
| Low HDL, % |  | 35.2% | (28.5%-42.6%) | 59.8% | (53.2%-66.1%) |  | 87.4% | (82.7%-91.0%) |
| Elevated BP, % |  | 17.4% | (12.8%-23.1%) | 51.5% | (46.8%-56.2%) |  | 55.4% | (46.9%-63.7%) |
| High fasting glucose, % |  | 10.1% | (6.0%-16.4%) | 48.4% | (41.1%-55.9%) |  | 63.9% | (55.1%-71.8%) |
| MetS, % |  | 5.9% | (3.4%-10.0%) | 61.5% | (56.7%-66.0%) |  | 93.8% | (89.6%-96.3%) |
| Diabetes, % |  | 0.7% | (.1%-4.3%) | 6.6% | (4.4%-9.6%) |  | 25.4% | (20.2%-31.5%) |
| Without NAFLD |  | True Negative | | | |  | False Positive | |
| Characteristics |  | Low LFS (N=2797) | | Intermediate LFS (N=1173) | |  | High LFS (N=147) | |
| AST/ALT ratio |  | 1.58 | (1.52-1.64) | 1.16 | (1.11-1.2) |  | 1.03 | (0.92-1.15) |
| Aspartate aminotransferase: SI(U/L) |  | 18.68 | (18.3-19.05) | 21.88 | (20.82-22.93) |  | 24.83 | (23-26.66) |
| Alanine aminotransferase: SI (U/L) |  | 13.45 | (12.93-13.96) | 21.31 | (20.41-22.2) |  | 27.46 | (23.67-31.25) |
| Serum insulin (uU/mL) |  | 7.01 | (6.88-7.13) | 12.50 | (12.2-12.8) |  | 27.01 | (25.06-28.96) |
| Abdominal_obesity, % |  | 17.5% | (16.2%-18.9%) | 54.0% | (50.0%-57.9%) |  | 83.5% | (69.4%-91.9%) |
| Hypertrigeridemia, % |  | 12.3% | (10.7%-14.0%) | 49.9% | (46.4%-53.5%) |  | 68.4% | (57.1%-77.8%) |
| Low HDL, % |  | 26.7% | (23.9%-29.7%) | 58.8% | (55.0%-62.5%) |  | 65.3% | (49.3%-78.4%) |
| Elevated BP, % |  | 13.90% | (11.8%-16.2%) | 43.20% | (39.3%-47.3%) |  | 62.90% | (51.3%-73.2%) |
| High fasting glucose, % |  | 10.2% | (8.5%-12.2%) | 45.8% | (42.2%-49.5%) |  | 61.7% | (47.8%-73.9%) |
| MetS, % |  | 3.1% | (2.4%-3.9%) | 52.6% | (48.6%-56.5%) |  | 77.1% | (66.7%-85.0%) |
| Diabetes, % |  | .1% | (.0%-.2%) | 5.0% | (3.8%-6.4%) |  | 27.5% | (20.5%-35.8%) |

Supplementary Table 4. Definition of NAFLD used in different NAFLD prediction score studies.

| NAFLD prediction scores | Imaging tool used | Definition of NAFLD | References |
| --- | --- | --- | --- |
| LFS | ^1^H-MRS | NAFLD was defined as liver fat 55.6 mg triglyceride/g liver tissue or 5.56% of liver tissue weight. | 1 |
| FLI | Ultrasonography | Steatosis was defined as the presence of an ultrasonographic pattern consistent with “bright liver,” with evident ultrasonographic contrast between hepatic and renal parenchyma, vessel blurring, focal sparing, and narrowing of the lumen of the hepatic veins, according to international guidelines | 2-3 |
| LAP | Ultrasonography | Hepatic steatosis was quantified with a method very similar to that recently validated by Hamaguchi et al. Normal liver was defined as the absence of liver steatosis or other liver abnormalities. Light steatosis was defined as the presence of slight “bright liver” or hepatorenal echo contrast without intrahepatic vessels blurring and no deep attenuation; moderate steatosis as the presence of mild “bright liver” or hepatorenal echo contrast without intrahepatic vessel blurring and with deep attenuation; and severe steatosis as diffusely severe “bright liver” or hepatorenal echo contrast, with intrahepatic vessels blurring (no visible borders) and deep attenuation without visibility of the diaphragm | 4 |
| HSI | Ultrasonography | Semiquantitative grading of fatty liver was done as described by Saadeh et al., as follows: grade 0, normal echogenicity; grade 1, slight, diffuse increase in fine echoes in liver parenchyma with normal visualization of the diaphragm and intrahepatic vessel borders; grade 2, moderate, diffuse increase in fine echoes with slightly impaired visualization of intrahepatic vessels and the diaphragm; grade 3, marked increase in fine echoes with poor or no visualization of intrahepatic vessel borders, the diaphragm, and the posterior right lobe of the liver. | 5 |

[1] Szczepaniak LS, Nurenberg P, Leonard D, et al. Magnetic resonance spectroscopy to measure hepatic triglyceride content: prevalence of hepatic steatosis in the general population. Am J Physiol Endocrinol Metab 2005;288:E462–E468.

[2] Saverymuttu SH, Joseph AE, Maxwell JD. Ultrasound scanning in the detection of hepatic fibrosis and steatosis. Br J Med Clin Res Ed. 1986; 292.13-5

[3] Joseph AE, Saverymuttu SH, al-Sam S, Cook MG, Maxwell JD. Comparison of liver histology with ultrasonography in assessing diffuse parenchymal liver disease. Clin Radiol. 1991; 43.26-31

[4] Hamaguchi M, Kojima T, Itoh Y, Harano Y, Fujii K, Nakajima T, Kato T, Takeda N, Okuda J, Ida K, Kawahito Y, Yoshikawa T, Okanoue T: The severity of ultrasonographic findings in nonalcoholic fatty liver disease reflects the metabolic syndrome and visceral fat accumulation. Am J Gastroenterol 2007, 102:2708-2715

[5] Saadeh S, Younossi ZM, Remer EM, et al. The utility of radiological imaging in nonalcoholic fatty liver disease. Gastroenterology 2002;123:745–50.

Supplementary Table 5. Association of various markers of NAFLD or NAFLD fibrosis with all-cause mortality.

| Prediction score of NAFLD/ fibrosis |  | HR | 95% CI | P value |
| --- | --- | --- | --- | --- |
| LFS (continuous) |  | 1.09 | (1.01-1.19) | 0.039 |
| FLI (continuous) |  | 1.00 | (0.99-1.01) | 0.602 |
| LAP (continuous) |  | 0.81 | (0.62-1.06) | 0.126 |
| HSI (continuous) |  | 0.98 | (0.94-1.01) | 0.118 |
| NFS (continuous) |  | 1.03 | (0.95-1.11) | 0.473 |

Model was adjusted for age, sex, race or ethnicity, education, income, diabetes, hypertension, history of CVD, lipid-lowering medication, smoking status, waist circumference, alcohol consumption, caffeine consumption, total cholesterol, high-density lipoprotein cholesterol, transferrin saturation, and C-reactive protein.

Supplementary Table 6. Association between LFS and cardiometabolic disease related mortality with adjustment of the Framingham Risk Score.

| Mortality |  | HR | 95% CI | P value |
| --- | --- | --- | --- | --- |
| CVD related |  |  |  |  |
| Low (ref) vs. High |  | 1.88 | (0.95-3.72) | 0.068 |
| Intermediate (ref) vs. High |  | 1.74 | (0.87-3.5) | 0.117 |
| LFS (continuous) |  | 1.08 | (1-1.17) | 0.046 |
| Liver disease related |  |  |  |  |
| Low (ref) vs. High |  | 14.93 | (5.65-40) | <0.001 |
| Intermediate (ref) vs. High |  | 4.29 | (1.7-10.87) | 0.003 |
| LFS (continuous) |  | 1.33 | (1.21-1.47) | <0.001 |
| Diabetes related |  |  |  |  |
| Low (ref) vs. High |  | 14.29 | (2.25-90.91) | 0.006 |
| Intermediate (ref) vs. High |  | 16.95 | (2.4-125) | 0.005 |
| LFS (continuous) |  | 1.24 | (1.13-1.37) | <0.001 |

Model was adjusted for sex, race or ethnicity, and the Framingham Risk Score for hard coronary heart disease*.

*the score was calculated based on age, total cholesterol, HDL, systolic blood pressure, Treatment for hypertension, and smoking status. Reference: Expert Panel on Detection, Evaluation, and Treatment of High Blood Cholesterol in Adults (2001) Executive Summary of The Third Report of The National Cholesterol Education Program (NCEP) Expert Panel on Detection, Evaluation, And Treatment of High Blood Cholesterol In Adults (Adult Treatment Panel III). JAMA, 285, 2486-2497.)

Supplementary Table 7. Area under ROC curve (AUC) and 95% CI of NAFLD prediction scores in predicting NAFLD that defined by “none (no NAFLD) vs. moderate-severe steatosis (NAFLD)” , “none (no NAFLD) vs. severe steatosis (NAFLD)”, and “none (no NAFLD) vs. mild-severe steatosis (NAFLD)”.

| NAFLD prediction scores | NAFLD (none vs. moderate-severe steatosis) | | NAFLD (none vs. severe steatosis) | | NAFLD (none vs. mild to severe steatosis) | |
| --- | --- | --- | --- | --- | --- | --- |
|  | AUC | 95% CI | AUC | 95% CI | AUC | 95% CI |
| LAP | 0.755 | (0.737-0.772) | 0.794 | (0.77-0.818) | 0.709 | (0.694-0.725) |
| HSI | 0.746 | (0.729-0.764) | 0.794 | (0.771-0.817) | 0.678 | (0.662-0.694) |
| FLI | 0.772 | (0.755-0.789) | 0.821 | (0.799-0.842) | 0.695 | (0.679-0.711) |
| LFS | 0.785 | (0.769-0.802) | 0.835 | (0.814-0.857) | 0.686 | (0.67-0.702) |

All p-value < 0.001

Supplementary Table 8. Association of mild hepatic steatosis with mortality.

| Mortality | HR | 95% CI | P-value |
| --- | --- | --- | --- |
| All-cause | 1.2 | (0.91-1.58) | 0.198 |
| Cardiovascular | 1.25 | (0.79-1.98) | 0.326 |

Model was adjusted for age, sex, race or ethnicity, education, income, diabetes, hypertension, history of CVD, lipid-lowering medication, smoking status, waist circumference, alcohol consumption, caffeine consumption, total cholesterol, high-density lipoprotein cholesterol, transferrin saturation, and C-reactive protein.

Supplementary Figure 1. Flow diagram of participants of the study


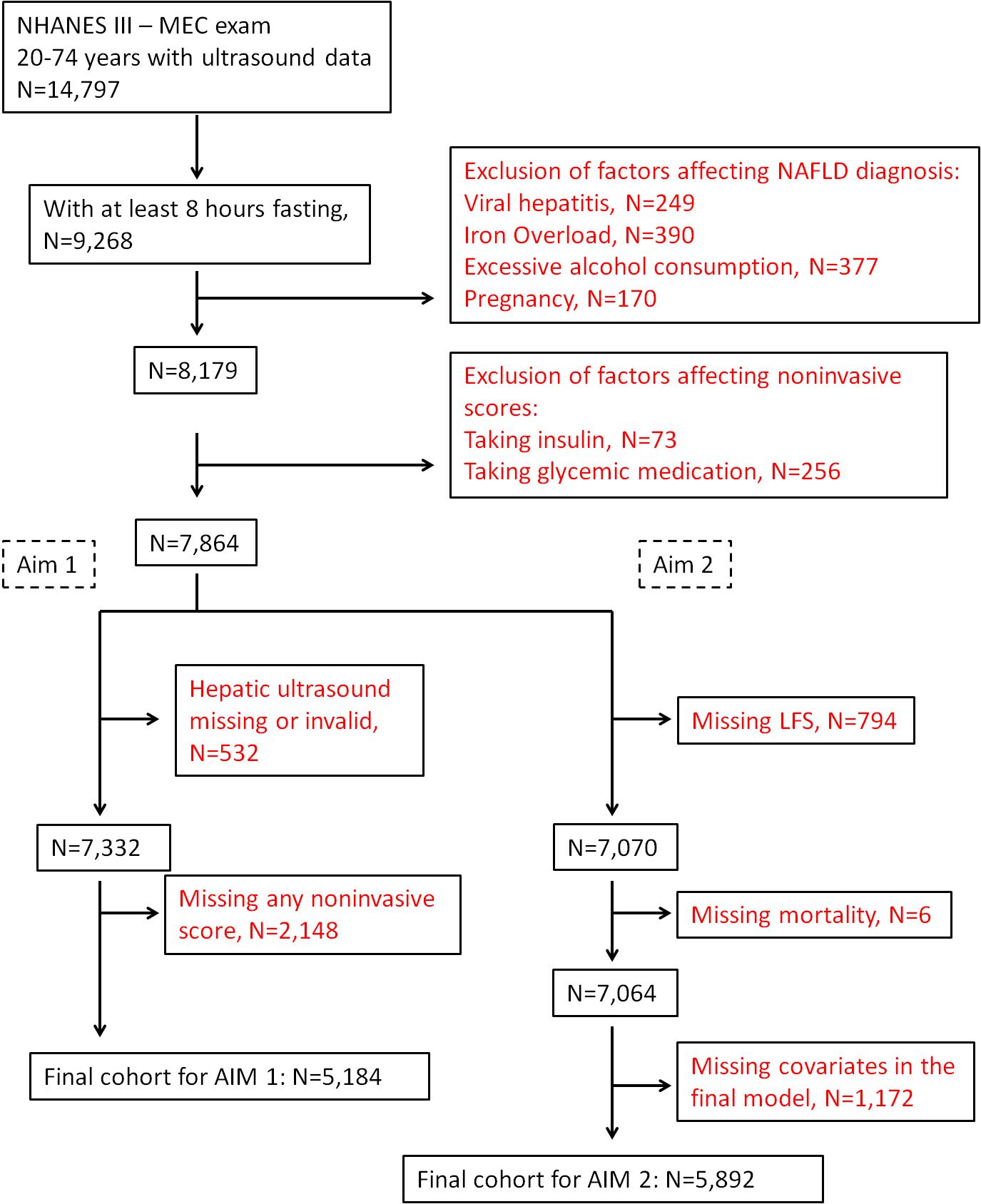

Supplement: Additional file 1: — Additional file contains supplementary Tables S1 to S8 and supplementary Figure S1. [file 12916_2014_154_MOESM1_ESM.docx]
